# Advances in Dietary Fiber: Classification, Functional Properties, Interactions, and Valorization of Dietary Fiber from Food Processing By-Products

**DOI:** 10.3390/foods15162826

**Published:** 2026-08-13

**Authors:** Xue Chen, Khalid Amin, Peng Liu, Xiaochun Yu, Hongling Fu, Ming Li

**Affiliations:** 1 College of Medicine and Health, Tonghua Normal University, Tonghua 134002, China; 13614352257@163.com (X.C.); liupeng9405@163.com (P.L.); yuxc@thnu.edu.cn (X.Y.); 2 College of Food Science and Engineering, Jilin Agricultural University, Changchun 130118, China; khalidscihn@gmail.com; 3 Division of Soybean Processing, Soybean Research & Development Center, Chinese Agricultural Research System, Changchun 130118, China

**Keywords:** dietary fiber, by-products, functional properties, gut health, food applications

## Abstract

During food processing, large quantities of by-products are generated, including seeds, fruit peels, pomace, shells, and other plant-derived residues. These by-products are usually rich in dietary fiber (DF) and possess significant potential for nutritional and functional applications. Dietary fiber plays an important role in maintaining human health and reducing the risk of cardiovascular disease, diabetes, and other non-communicable diseases. At the same time, the incorporation of dietary fiber into food systems can effectively improve the physicochemical properties of foods and enhance their functional characteristics. This article reviews the research progress on dietary fiber derived from food processing by-products, with a focus on the interactions between dietary fiber and other functional components in foods, as well as its potential effects on human health. In addition, this article summarizes and analyzes the current applications of food processing by-product-derived dietary fiber in traditional food processing, functional foods, and other emerging food-related fields and discusses its future development directions. This review aims to provide a theoretical reference for the high-value utilization of dietary fiber derived from food processing by-products. This review emphasizes that dietary fiber derived from different food processing by-products exhibits diverse compositional characteristics and functional potential, and its functional properties are closely associated with the original sources and characteristics of the raw materials. Reasonable utilization of food processing by-products can provide new avenues for the development of high-value functional food ingredients and sustainable food materials.

## 1. Introduction

The global food processing industry generates a large amount of by-products such as bran, fruit residue, vegetable leaves, and soybean residue during the processing of raw materials such as grains, fruits, vegetables, legumes, and other plant-based food materials [1]. These by-products are usually rich in dietary fiber (DF), making them valuable sources of functional dietary fiber with potential applications in food and health-related industries [2]. However, most by-products are directly discarded, lacking proper utilization, resulting in resource waste and environmental pollution. Dietary fiber has multiple physiological functions such as regulating intestinal health and improving metabolism. Extracting DF from waste and adding it to foods such as bread, biscuits, yogurt drinks, etc., can not only optimize the processing performance of food and enhance its health benefits but also further realize the high-value utilization of waste resources, thereby achieving a dual transformation from environmental burden to nutritional gain. Therefore, how to efficiently utilize these by-products has become an important issue that needs to be addressed for the sustainable development of the food industry.

Dietary fiber is an indigestible carbohydrate that cannot be hydrolyzed or digested by the human small intestine, while it can be fully or partially fermented in the large intestine [3]. It can be defined as “the indigestible carbohydrates and lignin inherent and intact in plants”. The World Health Organization (WHO) and the Codex Alimentarius Commission defined DF as “a molecule with 10 or more structural units in its structure”. Recently, the International Carbohydrate Quality Alliance referred to DF as “non-absorbable plant carbohydrates”. DF can be divided into two categories according to their solubility, which are soluble dietary fiber (SDF), based on pectin, vegetable gums, β-glucan, galactomannan, and fructan, and insoluble dietary fiber (IDF), which is hemicellulose and cellulose that cannot be digested and absorbed for energy metabolism. DF has been regarded as a non-nutritious substance for a long time due to its unique physicochemical properties, but with the continuous progress of nutritional science research, the important role of DF has been affirmed and it has been hailed as the “seventh major class of nutrients” [4].

In recent years, the resource utilization of food processing by-products has been increasingly valued, especially the DF derived from these by-products, which has become a research hotspot in the food industry’s dietary supplements due to its resource regeneration and functional superiority, demonstrating wide application potential. Existing research not only focuses on exploring the functional characteristics of such fibers but also actively explores their potential applications in material development and healthy food. Scarcella et al. successfully developed a biodegradable SDF film with good mechanical properties using soybean residue, which can rapidly degrade in natural environments and provide an environmentally friendly new solution to replace traditional plastic packaging [5]. Cao et al. (2024) compared the physicochemical and biological activities of SDF in the by-products of *Saccharina japonica* and found that SDF showed better performance in water retention, swelling, ion exchange, and antioxidant properties. It also demonstrated its ability to alleviate colitis by regulating oxidative stress and inflammatory response, providing a new pathway for functional food intervention in intestinal diseases [6]. Yu et al. focused on processing modification technology and found that the structural characteristics, physicochemical properties, and functional activity of soybean residue DF, which was treated with 1% K_2_CO_3_ combined with heat treatment and hydrolysis modification, were optimized, providing a technical basis for its precise application in intestinal health food [7]. Even some discarded DF sometimes exhibits better characteristics than DF in food, further highlighting the enormous potential of converting such waste resources into high value-added products.

Compared with extracting dietary fiber from whole plant-based foods, using food processing by-products as a source of DF offers significant and scalable advantages. These residues are continuously generated in large quantities during food processing, are available at relatively low cost, and serve as rich sources of dietary fiber without requiring additional land or agricultural inputs. Moreover, their valorization is consistent with circular economy principles. Although several reviews have summarized dietary fiber from different perspectives, their focuses have mainly been limited to extraction technologies, physicochemical properties, functional modifications, or the health-promoting effects of dietary fiber from conventional plant sources. Some studies have discussed dietary fiber from individual food processing residues; however, a comprehensive overview of waste-derived dietary fiber as a sustainable resource remains limited. Unlike previous reviews, this work emphasizes the conversion of food processing by-products into value-added dietary fiber, focusing on its structure–function relationships, health effects, and high-value applications. At the same time, the application status of waste source DF in cutting-edge fields such as traditional food improvement, functional food development, and new food materials was summarized and evaluated, and its future development direction and potential path in the food industry were discussed. This study aims to provide a theoretical basis and technical reference for the systematic extraction and high-value utilization of waste source DF, in order to promote the transformation of the food industry towards a resource efficient and environmentally friendly sustainable model.

## 2. Literature Search on Dietary Fiber and Research Hotspots on Waste Dietary Fiber

**Figure 1:**
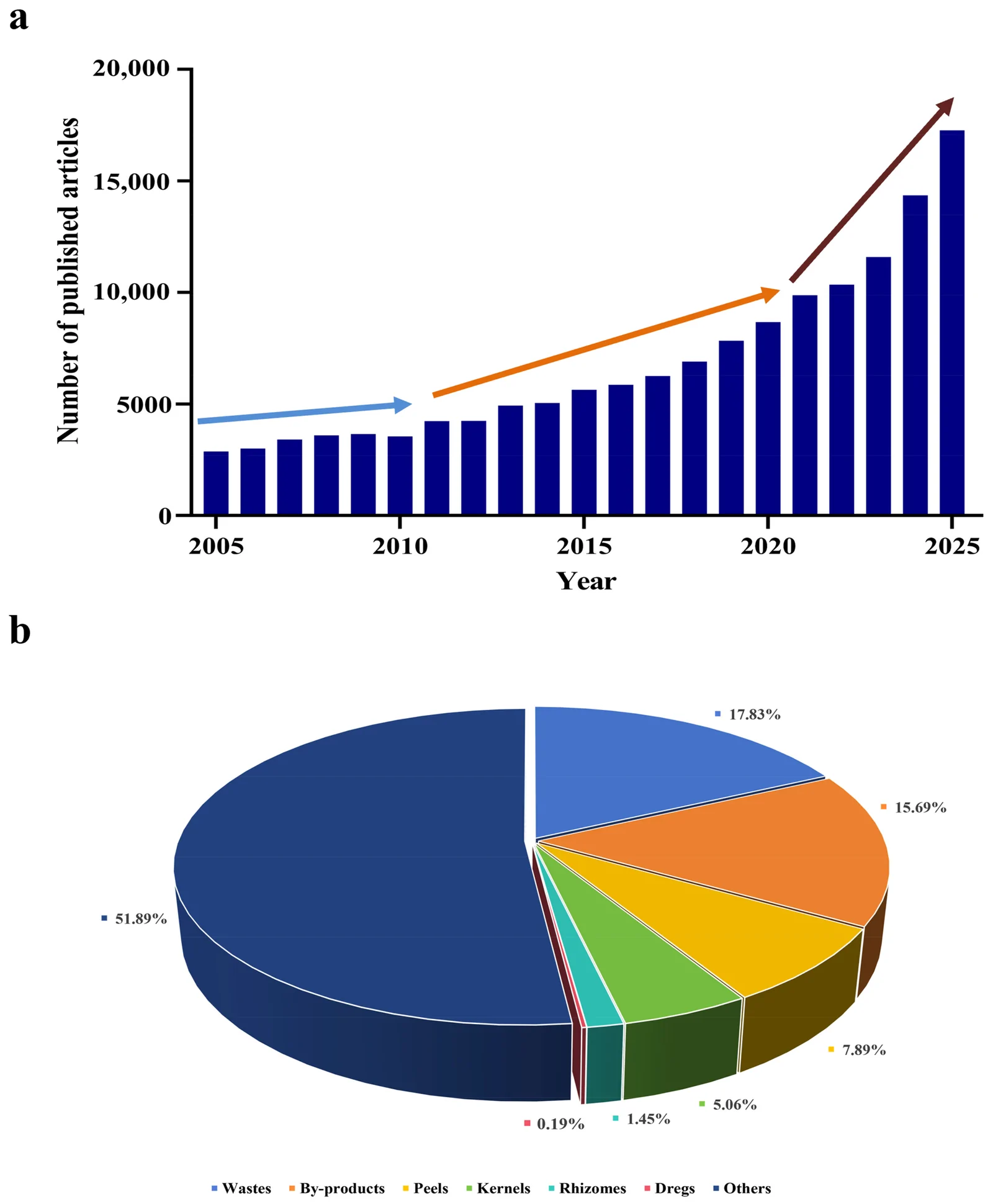
Statistical analysis of current research status of dietary fiber and waste dietary fiber. (**a**) The number of studies related to dietary fiber each year from 2010 to 2025 shows interest in DF. The blue, orange-brown, and dark-brown arrows indicate slow, moderate, and rapid growth in publication output, respectively. (**b**) Statistical analysis table of the proportion of research on waste dietary fiber.

Using “dietary fiber” as the primary search term, we retrieved 190,322 relevant articles published between 2010 and 2026 from the ScienceDirect database.Due to the unavailability of the complete annual dataset for 2026, the data for that year is not included in the visualization results of Figure 1a. Figure 1a shows that the number of relevant studies increased annually from 2010 to 2025, indicating the growing research interest in DF. The keywords used to retrieve dietary fiber from waste sources are composed of "Dietary Fiber" combined with various source terms, including wastes, by-products, peels, kernels, rhizomes, dregs. Based on the statistical results presented in Figure 1b, the number of studies focusing on waste dietary fiber accounted for less than 50% of all DF-related studies. Overall, research on dietary fiber has received increasing attention, while research on waste dietary fiber remains relatively limited. Therefore, this review systematically summarizes recent advances in dietary fiber, including its classification, functional properties, processing strategies, interactions with other components, and applications, and the potential for the resource utilization of waste dietary fiber from food processing by-products. The literature discussed in this review was selected and filtered based on its relevance to dietary fiber classification, functional properties, processing strategies, interactions with other components, and applications and waste dietary fiber utilization. Studies unrelated to these topics were not considered in the review.

**Figure 2:**
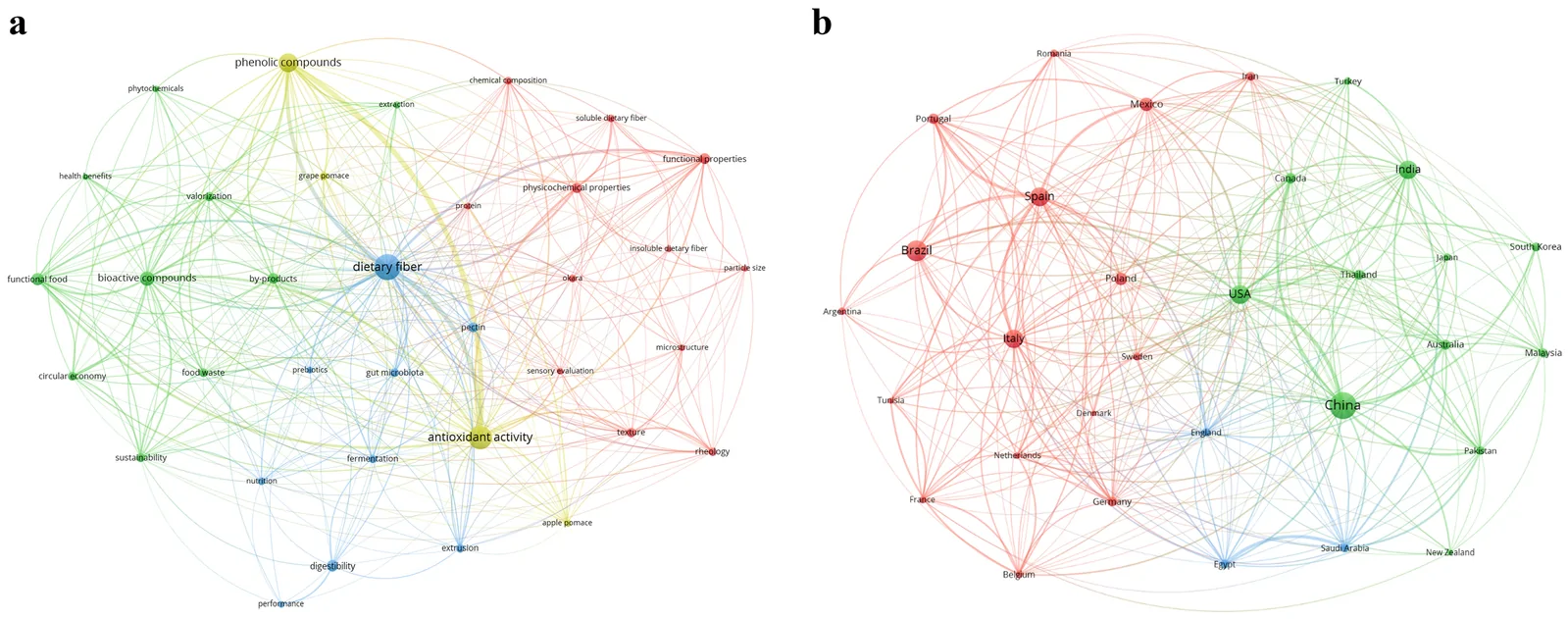
Bibliometric analysis of waste-derived dietary fiber based on the Web of Science database. (**a**) Keyword co-occurrence analysis of waste-derived dietary fiber using VOS viewer 1.6.21. Four clusters were identified, namely physicochemical and functional properties of raw materials (red area), resource valorization of by-products (green area), digestion and gut nutrition (blue area), and phenolic compounds and antioxidant activity (yellow area). (**b**) Country distribution analysis of publications related to waste-derived dietary fiber. Countries with a minimum of 50 documents were included, and China, the United States, and Spain showed relatively higher research outputs.

Through the Web of Science database, 3096, 1802, 1390, 468, 36, and 22 articles were retrieved using keywords “dietary fiber” and various by-products such as “by products”, “wastes”, “peels”, “kernels”, “rhizome”, and “dregs”, respectively. All articles were imported into the bibliometric analysis tool VOSviewer (Version 1.6.21) for analysis. Setting the minimum occurrence frequency of keywords to 40, out of 9302 related keywords, 48 keywords reached this threshold. After merging keywords with repeated meanings, 35 keywords were ultimately retained for subsequent mapping. As shown in Figure 2a, four clustering groups are obtained, representing four major research directions: physicochemical and functional properties of raw materials (red area), resource valorization of by-products (green area), digestion and gut nutrition (blue area), and phenolic compounds and antioxidant activity (yellow area). By analyzing the detailed distribution of each clustering module, we find that the red area focuses on raw materials of dietary fiber by-products, mainly involving classification (SDF/IDF), chemical composition, microstructure, particle size, physicochemical properties, rheological and textural properties, as well as sensory evaluation. The green area concentrates on agricultural by-products, mainly studying extraction, bioactive compounds, phytochemicals, functional food, health benefits, and also covers food waste utilization, valorization, sustainability and circular economy. The blue area takes dietary fiber as the research object, focusing on extrusion, fermentation processing technologies, as well as pectin, digestibility, prebiotic effects, gut microbiota and performance. The yellow area mainly takes fruit pomaces such as apple pomace and grape pomace as research objects to investigate phenolic compounds and their antioxidant activity. At the same time, based on the country as the analysis unit, the minimum number of documents required for a country is set at 50, and a total of 32 countries have reached this threshold. As shown in Figure 2b, the top three countries with relatively more literature on dietary fiber waste are China, the United States, and Spain. The search results indicate that China, the United States, and Spain have significant influence and contributions in the field of waste dietary fiber research and are some of the world’s important research forces in this field. Therefore, based on the hot structural content revealed by bibliometric analysis, we summarized the classification, structure, and properties of dietary fiber; emphasized its interactive application with prebiotics and phenolic substances; and focused on analyzing the health benefits of dietary fiber. Finally, we focused on the perspective of circular economy and resource utilization to summarize and analyze the application status and problems of waste DF in traditional food processing, health food, and cross functional food fields in today’s market and provided ideas for further processing DF in waste. The identified keyword clusters reflected the main research directions of waste dietary fiber and were consistent with the topics discussed in this review.

## 3. Food Processing By-Products as Sources of Dietary Fiber

Food processing by-products contain abundant dietary fiber (DF), whose composition and functional characteristics vary significantly depending on the source of raw materials and processing. According to its water-soluble characteristics, DF is usually divided into soluble dietary fiber (SDF) and insoluble dietary fiber (IDF). The proportion and composition of SDF and IDF in different sources of food by-products vary, which in turn affects their physicochemical properties, physiological functions, and potential applications in the food industry. For example, by-products of fruit and vegetable processing are often rich in SDF such as pectin, while plant residues such as grain bran and soybean residue contain a higher proportion of IDF such as cellulose and hemicellulose. Therefore, clarifying the composition characteristics and functional differences in DF from different by-product sources is an important foundation for achieving the high-value utilization of food processing waste. Although dietary-fiber-derived materials have also been explored in other fields, the applications discussed in this section mainly focus on food-related uses, including as functional food ingredients and food packaging materials. The two have significant differences in their structure, physicochemical properties, and physiological functions. SDF, such as pectin and β-glucan, has outstanding effects in regulating glucose and lipid metabolism and improving gut microbiota due to its good solubility and fermentation characteristics. IDF, mainly composed of cellulose, hemicellulose, and lignin, although not broken down by human digestive enzymes, is expanding from its traditional role as an intestinal regulator to a high-potential functional biobased material in the green transformation of the food industry due to its unique physical structure and material properties. In-depth research on the components of DF is not only related to the exploration of its nutritional value but is also the key to achieving the high-value utilization of agricultural and food processing by-products and developing new food materials.

### 3.1. SDF

Multiple polysaccharides are involved in the DF of waste sources, including pectin, konjac glucomannan, inulin, and other functional polysaccharides. Pectin mainly comes from fruit processing residues such as orange peel and apple pomace, while konjac glucomannan and inulin come from konjac tubers and plants rich in inulin, respectively. Chitin and Chitosan are polysaccharides from marine sources, often studied as functional components due to their unique physicochemical properties. Pectin is a complex structure of polysaccharide macromolecules and is widely found in the cell walls of terrestrial plants. It accounts for the highest amount of water-soluble DF in nature. Pectin holds strong medicinal value in the field of medicine due to its pharmacological and nutraceutical effects, such as antibacterial, detoxification, anticancer, lipid-lowering, and cholesterol-lowering capacities. Pectin was found to mediate host glucose regulation via gut flora [8]. Pectin extracted from dragon fruit skin can be used to prepare biodegradable films with good thermal stability and mechanical properties [9]. Pectin extracted from the peel of passion fruit may also hold promise for use as a thickener and gelling agent [10].

As a DF, β-glucan is a linear macromolecular polysaccharide composed of D-glucose monomers linked by β-glycosidic bonds, which is widely found in plants and microorganisms [11]. A large number of studies have proved that β-glucan has a variety of physiological functions and has good prospects for application in the food and pharmaceutical industries. As a new type of functional food, regular consumption of β-glucan can regulate blood glucose and lower blood lipids and plasma cholesterol, which leads to alleviating obesity and improving the overall efficacy of diabetes mellitus (DM) treatment conditions. β-glucan can also improve the immune response of the organisms and has a certain degree of antitumor activity [12]. β-glucan has been used as a functional food additive in milk, yogurt, and bread to reduce energy intake and lower cholesterol.

Konjac glucomannan (KGM) is a naturally occurring SDF. It was found that the use of KGM in mice (with ionizing radiation-induced damage to the hematopoietic and gastrointestinal systems) enhanced the abundance of intestinal microbiota, the number of probiotic organisms, and the production of short-chain fatty acids (SCFAs), which maintained intestinal homeostasis and significantly suppressed apoptosis of irradiated human intestinal epithelial cells [13]. Functional inulin is another SDF that possesses a wide range of biological activities, including immunomodulatory, antioxidant, antitumor, hepatoprotective, hypoglycaemic, and gastrointestinal protective activities [14,15].

Inulin-type fructans (ITFs) are dietary prebiotic fibers that can have beneficial health effects with the potential to alter the gut microbiota [16]. Different classes of SDF from the same foodstuffs tend to exhibit different physicochemical properties. Therefore, research on the functional and physicochemical properties of SDF in foods need to be further explored, deepened, and strengthened. At present, SDF is more widely used in food, such as in the regulation of obesity as a functional food raw material, the bacteriostatic preservation effect of edible film, and so on.

However, as the content of SDF in food is often much lower than that of IDF, many scholars have devoted themselves to exploring the modification strategies of DF to obtain more SDF, but the results are often not significant enough. Constrained by the DF content, the extraction of SDF from foodstuffs themselves as raw materials for application in foodstuffs suffers from the disadvantages of low extraction rates and high economic costs. Therefore, the development and application of homologous food waste with high DF content has considerable practical and economic value.

### 3.2. IDF

IDF mainly consists of cellulose, hemicellulose, and lignin, which are important structural components of plant cell walls. Cellulose is a linear polymer IDF consisting of β-D-glucopyranose structural units linked by β-1,4-glycosidic bonds. It is widely distributed in nature and is the most abundant renewable polysaccharide in nature, which can be isolated and obtained from many plants and is a renewable and biodegradable natural polymer. Through mechanical, chemical, or enzymatic treatment, cellulose can be converted into nanocellulose with nanoscale and enhanced functional properties, bringing new opportunities for food-related applications. Nanocellulose has been investigated for diverse applications ranging from food packaging to biomedical materials because of its high specific surface area, sustainability, and environmental friendliness [17]. In the food sector, nanocellulose has good application prospects in food additives, functional factor delivery materials, food coatings, food packaging, and other fields due to its superior performance and characteristics.

Separating lignin-containing cellulose nanocrystals (LCNCs) from pineapple peel residue, a by-product of pineapple processing, and combining it with tannic acid (TA) through hydrogen bonding interactions forms LCNC-TA composite particles that can be used as a stable Pickering emulsion, which is important for the high-value utilization of lignocellulosic agricultural wastes [18]. Grafting cellulose nanocrystals (CNCs) prepared from buckwheat bran with curcumin in the form of ester groups enhances the antioxidant activity of gel particles and nanoparticles. At the same time, nanocrystalline gel particles and nanoparticles can inhibit fat hydrolysis and digestion, thereby reducing the body’s absorption of fat and significantly lowering elevated levels of triglycerides, body weight, and fat accumulation [19]. Hemicellulose is the second-largest carbohydrate component of primary and secondary cell walls after cellulose. Hemicellulose is widely distributed in lignocellulosic materials, including cereals, fruits, vegetables, legumes, and other plant-derived agricultural by-products, where it represents one of the major structural polysaccharides and contributes substantially to dietary fiber. The main types of hemicelluloses encountered in diets are xylan, galactomannan, glucomannan, and xyloglucan. Compared with other IDF, the structure of hemicellulose is more diverse and complex. There are a large number of hydroxyl groups in the structure of hemicellulose, which can be crosslinked or chemically modified to prepare materials with different functionalities, which can be applied in the fields of drug delivery, tissue engineering, and wastewater treatment. Several kinds of hydrogel adsorbents can be made of hemicellulose for the removal of metal ion contaminants such as Cd (II), Cu (II), and Pb (II) from aqueous solutions. Xylan hemicellulose can be used as a renewable material for food packaging applications. Xylan-coated film samples showed good performances in terms of water, air, and water vapor permeability; solubility; mechanical strength; and antimicrobial activity against pathogenic bacteria [20]. Lignin, which is insoluble in water and indigestible by the human body, is a component of IDF and is considered one of the main components of plant cellulosic biomass. Lignin is an integral part of the secondary cell wall of the plant, which fills in the spaces between the cellulose and gives the plant its rigid structure. The high carbon content and phenolic structure of lignin make it water-resistant, solvent-resistant, flame-retardant, aging-resistant, light-absorbing, etc., which together determine the excellent properties of lignin as a composite material. Lignin has good antibacterial activity, and the anti-free radical and antibacterial activities of lignin and lignin-based films are important for applications such as food packaging additives. Lignin–chitosan biocomposite membranes were used for the preparation of active antimicrobial biofilms to effectively inhibit specific pathogenic and spoilage microorganisms [21]. Interest in IDF research once declined, but in the recent years, as scholars continue to deepen the study of IDF, understanding of the function of IDF has become more and more comprehensive. More and more of the efficacy and importance of IDF have been discovered and exploited. The IDF in some foods is even more effective than the SDF of the same food in lowering lipids and glucose. The expansion and application of IDF in food processing have been constrained by the fact that the addition of IDF can cause quality problems such as low organization, loose structure, and roughness in texture. However, as people continue to study and optimize the addition of IDF to food, many of the shortcomings in the food industry have been overcome and there have been better prospects for research and development.

In summary, the functional value of dietary fiber has more potential for differentiation due to the different types of soluble dietary fiber (SDF) and insoluble dietary fiber (IDF). SDF, due to its excellent solubility and fermentation characteristics, exhibits superior physiological activity in regulating glucose and lipid metabolism, improving intestinal microbiota and intervening in specific diseases. It is an important ingredient worth paying attention to in the development of functional foods. IDF not only has traditional physiological regulatory functions but has also become a potential raw material for developing new biobased food materials and promoting high-value resource utilization due to its structural stability and modifiability. The targeted extraction and utilization of SDF and IDF from food processing by-products can not only effectively enhance the nutritional value and material potential of waste resources but also provide diversified solutions for the food industry from functional ingredients to green packaging materials. Future research needs to further clarify the structure–activity relationship between the specific structures and functions of dietary fiber from different sources and focus on the development of green and efficient extraction and modification technologies, in order to fully tap into the application potential of dietary fiber and promote its systematic value in enhancing human health and sustainable development of the food industry.

## 4. Functional Properties of DF

The composition and structure of DF have a significant impact on its physicochemical and functional properties. A large number of experimental data have shown that the physicochemical properties and physiological functions of DF are closely related to its water-holding capacity, oil-holding capacity, glucose adsorption capacity, and cholesterol adsorption capacity, and the adsorption capacity of other harmful substances [22]. In food research and development (R&D) and industrial production, processing, and modification, different methods are used to change the structure of DF to optimize and improve its physicochemical and functional properties.

Currently, there are many methods for modifying DF, including physical, chemical, and biological, as well as composite modification methods that combine two or more of these methods. Since the SDF content in daily food is very low, DF modification can be used to increase the SDF content and improve its physicochemical properties to achieve a balanced DF profile and enhance its physiological effects on the human body. Twin-screw extrusion combined with ultrasonic treatment and enzymatic modification was used to treat DF from corn bran, and it was found that the extrusion–enzyme synergistically modified soluble dietary fiber (EESDF) exhibited a lower molecular weight; a higher extraction rate, water-holding capacity, and nitrite ion adsorption capacity; and higher antioxidant activity [23]. SDF from garlic peel modified by a twin-screw extrusion–cooking process has also been shown to have a positive effect on lead (Pb) binding [24]. However, it has also been shown that IDF has many physiological functions as well [25]. Highly purified insoluble dietary fiber (HPIDF) from soya bean dregs obtained using a complex enzymatic method had better physicochemical properties and better in vitro antioxidant activity, such as higher viscosity, oil-holding capacity, better cation exchange capacity, and α-amylase activity. Due to the small particle size and large specific surface area of HPIDF, the adsorption of heavy metals (Cd^2+^, Pb^2+^, Zn^2+^), glucose, cholesterol, and acrylamide by HPIDF is ideal [26]. Insoluble dietary fiber from Eucheuma (EIDF) has a strong hydroxyl radical scavenging ability, and the scavenging rate of EIDF obtained from several types of ultra-micro pulverization was higher than 97% and showed strong adsorption properties for aflatoxin B1 [27]. IDF extracted from both soursop (Annona muricata) and mango also showed good antioxidant activity [28].

However, the extraction and application of DF still face many challenges, such as low extraction efficiency, poor quality, instability, and low industrial production. Therefore, the extraction and optimization of DF, especially waste dietary fiber, is particularly important to further enhance the development value of DF and its application in the food and pharmaceutical industries.

## 5. Interactions of DF with Other Substances

**Figure 3:**
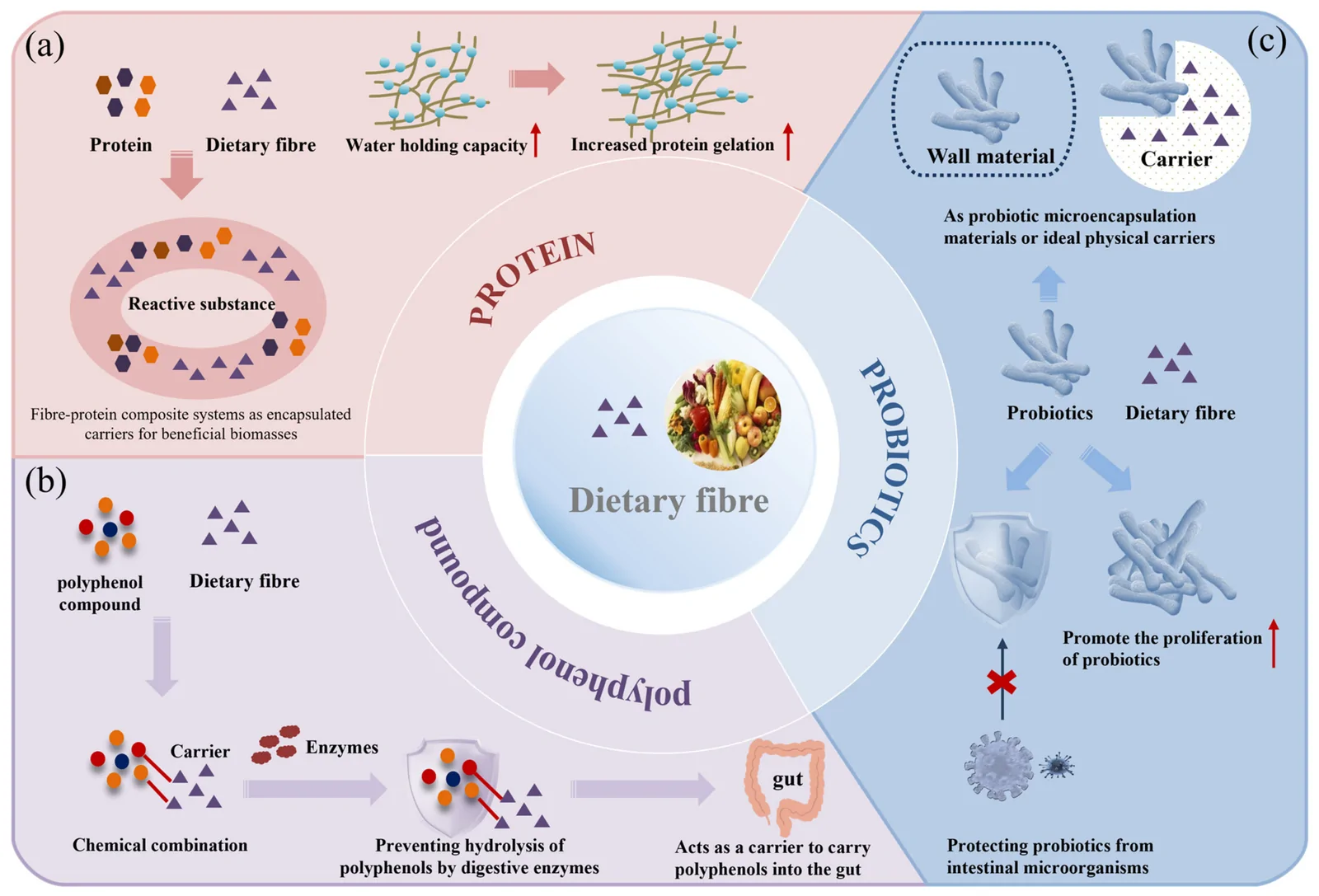
Interactions between dietary fiber and other bioactive components, including proteins, phenolic compounds, and prebiotics. (**a**) Interaction of DF with proteins, including binding interactions and their effects on protein stability and functional properties. (**b**) Interaction of DF with phenolic compounds, including adsorption, protection, and regulation of phenolic bioavailability. (**c**) Interaction of DF with prebiotics, illustrating the role of DF in modulating gut microbiota and promoting beneficial microbial activity.

For a long time, the health benefits of dietary fiber (DF), such as increasing satiety, regulating intestinal peristalsis, and adsorbing bile acids, have mainly been attributed to its own physical and chemical properties. However, modern nutritional research has shown that DF does not act in isolation within the human body. As a key component of complex foods such as whole grains, vegetables, fruits, and soy products, DF has a certain degree of mutually beneficial effects with coexisting phenolic compounds, proteins, probiotics, and other bioactive ingredients. These interactions are dynamically correlated during food digestion and together form a health regulatory network. The mechanism and effects of their mutual cooperation are shown in Figure 3, which systematically explains the multi-path and integrated health-promoting effects of dietary fiber when coexisting with phenols, proteins, and prebiotics.

### 5.1. DF Binding to Phenolics

Polyphenols are secondary metabolites containing more than 8000 compounds, which are very common in plants and are widely found in the vegetables, fruits, and spices of many kinds of plants. Polyphenols can be classified as flavonoids, phenolic acids, stilbenes, lignans, and ellagic acids. Because polyphenols and DF are often found in the same foods and are usually ingested together, research on the synergistic effects of DF and polyphenols on gut flora has continued to increase in recent years. Because both enter the colon at a similar time, certain dietary polyphenols are readily metabolized through the gastrointestinal tract and colon, undergoing structural modification or degradation before being absorbed by specific organs. In plant foods, polyphenols are predominantly covalently bound to DF, and the bioavailability of polyphenols often depends on the extent to which they are released from DF during digestion.

Dietary fiber (DF) may also play an important role in the metabolism and bioactivity of flavonoids by regulating intestinal flora [29]. For example, tea residue dietary fiber (TRDF) is rich in bound polyphenols. The synergistic interaction between its dietary fiber fraction (TRDF-DF) and bound polyphenol fraction (TRDF-BP) can regulate intestinal bacterial dysbiosis and increase the production of short-chain fatty acids (SCFAs) by enriching beneficial bacteria and inhibiting harmful ones. This synergy ultimately exerts a hypoglycemic effect, thereby enhancing the overall anti-hyperglycemic activity of TRDF [30]. In the synergistic use of a complex lotus root SDF and two phenolics, it was found that chemical binding between SDF and phenolics could take place. After the bonding of SDF and phenolics, the monosaccharide composition and molecular weight distribution of SDF changed significantly. Furthermore, the surfaces of the SDF–phenol complexes were aligned sparsely and uniformly, and both complexes showed higher thermal stability [31]. Insoluble dietary fiber rich in bound polyphenols (BP-IDF) was prepared from quinoa, rye, and wheat. It was found that all three whole-grain fiber-bound polyphenols improved the scavenging of carbonyl compounds during in vitro digestion and fermentation. BP-IDF exhibited the highest carbonyl scavenging capacity [32]. In summary, DF acts as a carrier to carry unstable polyphenols into the intestine, and the combination of DF and polyphenols can better protect the polyphenols from being hydrolyzed by digestive enzymes and promote the bioavailability of polyphenols in the digestive tract.

### 5.2. Effect of DF on Protein

When used synergistically with proteins, DF can affect the chemical forces between proteins, thereby improving some of their physicochemical properties. For example, the insoluble dietary fiber from Lentinus edodes stipe (LESIDF) promotes the transition of β-sheets to α-helices in the protein secondary structure and enhances the formation of ionic, hydrogen, and disulfide bonds, thereby maintaining a compact and stable 3D gel network [33].

It was found that when soybean hull complex DF (rice DF, soy DF, and inulin) was added to meat products as a fat substitute, the complex DF could influence the network structure of myofibrillar protein (MP) gels and improve their water retention and gel strength [34]. The same results were found in the investigation of the effect of sugarcane insoluble dietary fiber (SIDF) on the water-holding capacity (WHC) of myofibrillar protein (MP) gels, with WHC increasing with increasing SIDF content [35]. Soybean DF can also be used as a nutritional supplement and gel enhancer for soy protein isolate (SPI) gels, with 10% DF100 contributing to increased gel hardness (137.61–148.86 g) [36]. Nuo, Z. et al., proved that highly soluble dietary fiber okara–egg tofu produced by the combined treatment of steam explosion and enzymatic hydrolysis improved gelling properties [37]. The effects of the appropriate addition of DF on the properties of protein gels and emulsification characteristics, among others, showed an overall favorable trend. However, it is important to note that excess DF can affect the cross-linking of protein molecules, thereby disrupting the textural properties of the gel product. Pepper essential oil (SPEO) has the disadvantages of high volatility, poor stability, and low water solubility when applied in food. To overcome the above-mentioned drawbacks of SPEO, a composite cohesive layer shell material SPI-SDF consisting of SDF from peppercorn seeds and soybean protein isolate (SPI) was developed by coagulation and spray-drying technology, which successfully improved the solubility and bioactivity and enhanced the antimicrobial activity [38]. Due to the wide variety of DF and their differentiated nature, there is a wider scope for the development of DF applications in protein products. Currently, more research has been done on the application of fiber–protein gel systems or fiber–protein–other-substances gel systems, which show good potential, especially as carriers for encapsulation and the controlled delivery of various bioactive substances. In addition, there are many effects of fiber–protein composite systems, such as improving the nutritional properties of meat products, which need to be further explored by researchers and scholars.

### 5.3. DF as Prebiotics and Synergy with Probiotics

Probiotics play an important role in the food and pharmaceutical industries and have a huge beneficial effect on regulating immunity, lowering cholesterol levels, improving lactose tolerance, and preventing certain cancers in human health. In recent years, the enhancement of probiotic production through the addition of growth-promoting substances such as DF is a new technology in the food industry. DF can also be used as a coating material for probiotic microcapsules or as an ideal physical carrier for probiotics, shielding them from gastric acid and bile salts during gastrointestinal digestion, and subsequently assisting them in outcompeting harmful intestinal microorganisms [39]. DF in combination with probiotics also has the effect of improving metabolic abnormalities. DF is considered a prebiotic for gut health and can be used as a carbon source to support the growth and colonization of probiotic bacteria such as bifidobacteria, thereby promoting the proliferation of probiotics [40]. Soybean hull insoluble dietary fiber extracted enzymatically was found to induce an increase in the proliferation of beneficial bacteria, especially bifidobacteria [41]. Red ginseng dietary fiber (RGDF) extracted from red ginseng dregs promotes the proliferation of Lactobacillus plantarum IDCC3501 [42]. Numerous in vitro studies have demonstrated the synergistic effects of DF and probiotics, especially their probiotic effects on gut microbes. There is still much room for exploration in different DF and prebiotic combination studies. It is therefore necessary to explore comparative studies on the effects of different combinations of several common DF and probiotics or comparative studies on the function of different DF as wall materials for delivering probiotics. DF with different structural properties have different effects on the proliferation of probiotics, but it is important to note that not all DF have beneficial effects on and value-add to probiotics, something that needs to be supported by a large amount of research data.

In summary, understanding the synergistic mechanism between dietary fiber and food components such as phenols, proteins, and probiotics is of great significance for developing the next generation of functional foods and formulating precise nutrition strategies. Dietary fiber is not only an important nutrient but also plays a synergistic role in the food matrix. These findings suggest that future food research and development can be based on multi-component composite systems such as “dietary fiber–polyphenol–protein” composite systems, the targeted design of products that combine nutrition, functionality, and good sensory quality, and can promote the development of personalized synthetic elements. Subsequent research can focus on elucidating the structure–activity relationship between different structural dietary fibers and specific components, verifying their health effects through multi-omics and clinical studies, and systematically evaluating the applicability of different dietary fibers as prebiotics or delivery carriers, thus providing a basis for constructing health intervention strategies based on overall food effects.

## 6. DF and Health

The health effects of waste-derived DF have been investigated through different levels of evidence, including in vitro studies, animal experiments, human clinical trials, and officially recognized health claims. However, the strength and reliability of evidence vary among different physiological outcomes and depend on specific DF sources and study designs. With the change in modern people’s diet structure and lifestyle, the prevalence of chronic diseases such as obesity, diabetes, cancer and intestinal diseases continues to rise. The incidence and development of these diseases are closely related to the imbalance of intestinal flora. The regulatory effect of dietary fiber on gut microbiota is crucial for maintaining the structural and functional stability of the intestinal environment. Insufficient intake of dietary fiber can lead to dysbiosis of the gut microbiota, which in turn affects the composition of microbial metabolites. Short-chain fatty acids are the most abundant microbial metabolites in the colon, mainly produced by the fermentation of prebiotics such as dietary fiber by gut microbiota. They play an important role in regulating immune cell function, maintaining intestinal homeostasis, and influencing the progression of various diseases.

**Figure 4:**
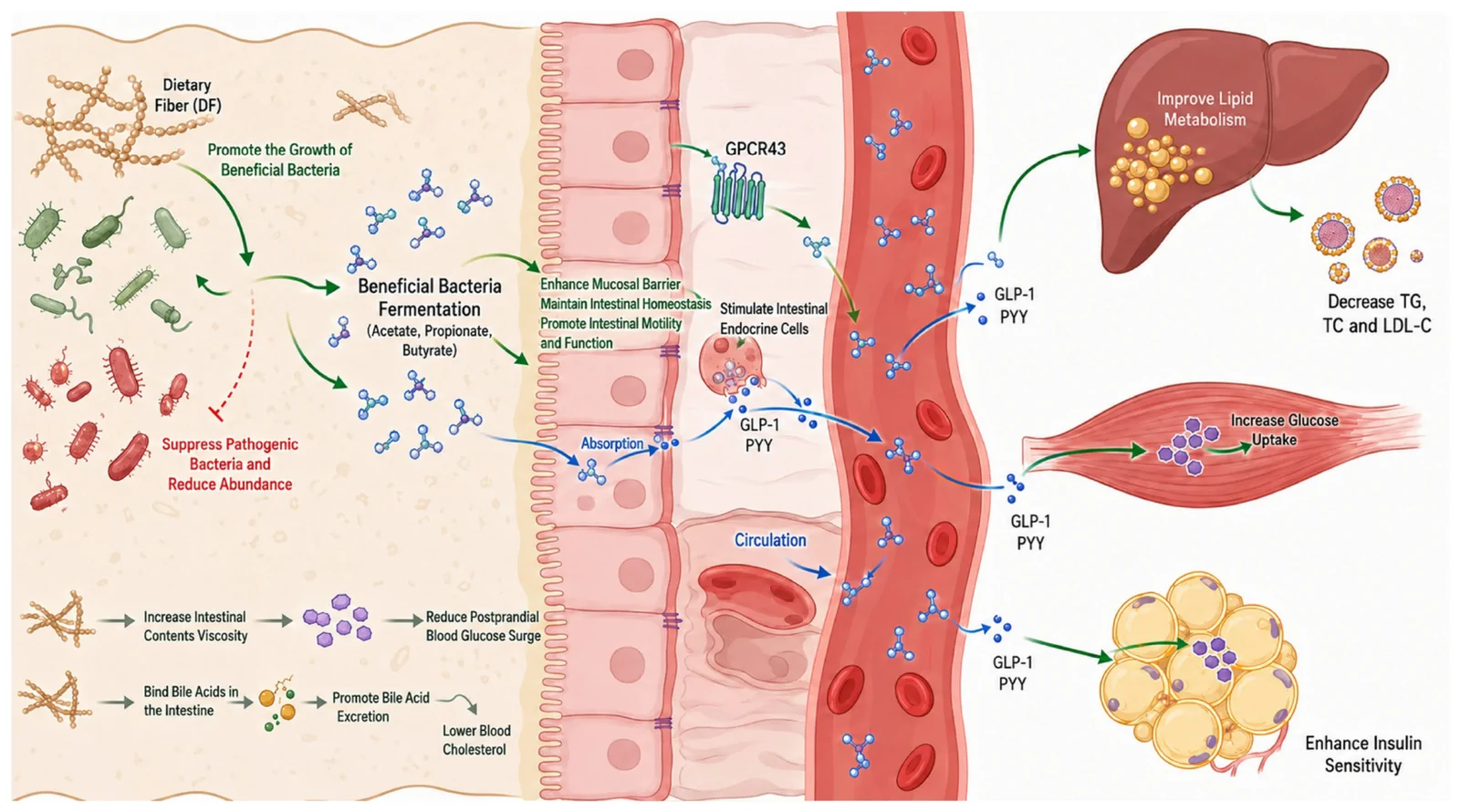
Regulatory effects of dietary fiber on gut microbiota and host health. Dietary fiber promotes the growth of beneficial bacteria and is fermented by gut microbiota to produce short-chain fatty acids (SCFAs), including acetate, propionate, and butyrate. SCFAs are absorbed by intestinal epithelial cells, enter circulation, and participate in the regulation of intestinal homeostasis and metabolic health.

DF induces changes in the production of SCFAs and in the composition of human intestinal flora [43]. It needs to be further investigated how DF regulates the metabolic and immune health of the host by interacting with the intestinal flora. The immune system is a barrier of organisms against various diseases, and immune response is one of the important biological activities of DF that enhance immune function. This makes DF a new agent to enhance immune potential. Growing body research suggests that regular intake of DF can help improve several conditions, such as controlling blood sugar, preventing constipation, improving high cholesterol, and, to some extent, alleviating atherosclerosis as well as reducing the risk of metabolic syndrome [44]. In the published Dietary Guidelines for Residents 2023, it is recommended that the diets of middle-aged and elderly people should focus on lowering the dietary inflammatory index by choosing foods rich in antioxidants, DF, and unsaturated fatty acids [45]. The main reason for this is the positive impact of DF on the prevention and management of several chronic diseases associated with aging, including diabetes, neurodegenerative diseases, cardiovascular diseases, and cancer [46]. The mechanism by which dietary fiber modulates intestinal flora to alleviate disease is shown in Figure 4. The specific positive effects of different sources and types of dietary fiber on different diseases are shown in Table 1.

### 6.1. DF and Gut Health

The human gut flora is a complex ecosystem consisting of trillions of bacteria, numbering approximately 1 × 10^14^ CFU, close to nearly 10 times the total number of cells in the human body, and is crucial for human immunity. DF can be fermented and broken down by the gut microbiota, and different sources of DF have different rates and degrees of fermentation. Irritable bowel syndrome (IBS) is one of the most common chronic intestinal disorders, characterized by recurrent episodes of gastrointestinal symptoms and a chronic functional gastrointestinal disorder characterized by abdominal pain and changes in bowel patterns.

There is growing evidence that DF can prevent and mitigate inflammatory bowel disease (IBD) by modulating the gut microbiota [61]. DF is fermented in the human gut to produce SCFAs, which act as a major energy and carbon source for gut microbes and play a key role in their colonization and growth of beneficial biomass, as well as in stimulating peristalsis and providing a variety of benefits.

The regulation of intestinal microecology is thought to encompass the following aspects: inhibition of intestinal pathogenic bacteria, promotion of intestinal probiotics, and enhancement of intestinal mucosal barrier function. SCFAs have been shown to increase the absorption of intestinal minerals, stimulate the growth of beneficial intestinal bacteria such as bifidobacteria, and increase the number of bacteria beneficial to bowel movements, as well as have some anti-inflammatory properties. It was found that HPIDF extracted from soya bean dregs could ameliorate the colonic environmental disturbances caused by acute ulcerative colitis to a certain extent. This effect is mainly associated with its influence on the proliferation of key bacteria in the synthesis pathway of SCFAs [26]. Barley leaf insoluble dietary fiber (BLIDF) reverses the DSS-induced decrease in SCFAs and secondary bile acids in mouse feces and exerts anti-inflammatory effects by modulating the composition of the intestinal flora and increasing the production of flora-derived metabolites [47]. The arabinoxylan in wheat has been shown to inhibit and reduce levels of the Th1 cytokine TNF-α, effectively preventing colitis by altering the intestinal microenvironment [62]. Rice hull insoluble dietary fiber (RHF) has also been found to be useful as a new dietary supplementation strategy against Cd-exacerbated colitis [48]. Psyllium DF has also been found to inhibit pro-inflammatory signaling, which may help prevent colitis [49]. Bamboo shoot DF alleviates colonic inflammation mainly by increasing the relative abundance of beneficial bacteria while decreasing the abundance of pathogenic bacteria (Escherichia-Shigella, Enterococcus, and Proteus) [50]. SDF from Lentinula edodes by-products may also have potential as a nutritional supplement to reduce colitis and increase by-product utilization [51]. Currently, the role of different types of DF in alleviating colitis is still being studied and explored, and the combined intake of specific DF and fat subtypes has also been suggested to have good efficacy in preventing colitis [26].

### 6.2. Hypoglycemic Effect of DF

In recent decades, the prevalence of DM has been increasing globally, and a series of complications caused by diabetes mellitus are also threatening the human health. DM is classified into four types: type 1, type 2, gestational, and other specific diabetes mellitus, with type 2 diabetes mellitus (T2DM) having the highest prevalence of up to 90%.

Currently, diabetes is regulated in several ways: first, by delaying intestinal glucose absorption; second, by promoting insulin secretion; and third, by increasing peripheral glucose utilization. It has been found that DF plays an active role in the prevention and treatment of diabetes, and a high-fiber diet can significantly improve insulin sensitivity, reduce insulin resistance, and slow down the rate of blood glucose increase [63]. The intake of DF can slow down the digestion of food, increase the viscosity of the intestinal fluid, and hinder the absorption of glucose [64]. The combination of DF and glucose helps to reduce the concentration of glucose, slowing down the rise in blood glucose and helping to control the level of blood glucose, thus preventing diabetes to a certain extent. DF can improve glucose blood lipid metabolism and hepatic dysfunction in diabetic patients and effectively intervene in the development of T2DM by altering the composition and structure of colonic flora in patients with T2DM. DF can also help T2DM patients by increasing their intestinal level of SCFAs, butyric acid, isobutyric acid, and isovaleric acid. It also improves insulin sensitivity, as a G protein-coupled receptor (GPCR) ligand, activates GPCR43 in the colon and influences glucose regulation by secreting hormones such as glucagon-like peptide-1 and tyrosyl Peptide YY (PYY), which enhance glucose uptake in muscle and adipose tissue. Studies have found that Bergamot DF can reduce serum total cholesterol, triglycerides, and LDL cholesterol and effectively alleviate diabetes mellitus (DM) [52]. Konjac glucomannan has also been found to be effective in alleviating hyperlipidemia and diabetes [53]. DF has also been found to be effective in controlling the gestational glycemic index in patients with gestational diabetes mellitus [65].

### 6.3. Anti-Obesity Effect of DF

In recent years, due to lifestyle changes, obesity has been increasing annually worldwide. Obesity, as a chronic metabolic disease, not only poses a threat to one’s health but is also one of the risk factors for the development of other diseases. Reducing the number of obese patients needs urgent approaches. It is particularly important to find an alternative treatment or prevention method that is organic, biocompatible, and non-toxic.

More and more studies have shown that there is a strong link between the dysbiosis of gut flora and obesity-related diseases. DF can play a key role in combating the development of obesity and can be effective in mitigating the side effects of obesity, and both the SDF and IDF have been shown to alleviate obesity [66]. DF can regulate food intake, digestion, absorption, and metabolism, thereby leading to the control of body weight [67]. Lipopolysaccharide-binding proteins are markers of systemic inflammation, and DF intake was found to be negatively correlated with lipopolysaccharide-binding proteins, which also indirectly led to reduced inflammation associated with obesity and metabolic diseases [68]. IDF from Okara has been found to prevent obesity by modulating hepatic mitochondrial fatty acid oxidative synthesis and degradation pathways [54]. The IDF in Okara can also be used in conjunction with intermittent fasting therapy to combat obesity synergistically by regulating the gut microbiota and its metabolites [69]. Pomelo peel dietary fiber can alleviate obesity in mice by improving intestinal flora dysbiosis [55]. Bamboo shoot dietary fiber (BSDF) may reduce obesity by improving the gut microbiota and modulating host PPAR and fatty acid metabolic pathways [56]. At present, there are a lot of studies on dietary fiber in lipid lowering and research on the lowering mechanism of each pathway is more and more in-depth, but the author believes that the joint application of multiple dietary fibers or synergistic research with other prebiotics should be increased to achieve synergistic effects. At the same time, safety standards should be established, as well as dosage recommendations and standards for appropriate populations.

### 6.4. Hypolipidemic Effect of DF

In some western developed countries, cardiovascular disease has become the leading cause of death. Lowering blood lipids and cholesterol levels to reduce the incidence of cardiovascular disease has become an urgent challenge for us to address. DF regulated the intestinal microbiota of mice fed with a high-fat diet; effectively inhibited TG, TC, LDL-C levels and the AI index; and improved the pathological abnormalities of the liver. DF from Tremella fuciformis residues pomace has also been shown to improve lipid metabolism disorders in hyperlipidaemic mice [57].

The synergistic effect of satsuma pomelo flavonoids (SPFEs) and dietary fiber (SPDF) increased the microbiota associated with the production of acetic acid and propionic acid, which synergistically alleviated hyperlipidemia. This hyperlipidaemic alleviation was achieved by modulating the production of butyric acid by the microbiota to modulate lipid metabolism and thus reduce blood lipids [29]. Bergamot DF showed potential for lowering blood cholesterol and body weight and could be used to develop new functional foods to prevent or treat obesity and hyperlipidemia [58]. DF can have a better cholesterol-lowering effect by regulating cholesterol metabolism and intestinal flora [70]. At the same time, DF also has good adsorption of cholesterol and can be combined with bile acids in the gastrointestinal system and transported out of the body as a waste product, thus reducing blood cholesterol levels [71,72]. Tea DF has been shown to improve serum and hepatic lipid distribution in mice fed a high-cholesterol diet [59]. Microbiologically fermented black rice DF improves cholesterol clearance by altering the composition of the intestinal microbiota (e.g., reduced Firmicutes and increased Akkermansia) [60].

### 6.5. DF and the Nervous System

Normal circadian rhythms play an important role in maintaining physiological and psychological health. Modern people’s poor dietary habits and late-night work and rest patterns can disrupt circadian rhythms, leading to difficulties in falling asleep, waking up frequently, and other sleep disorders. Several studies have shown that DF can play a beneficial role in sleep and mental health by regulating intestinal flora. The brain–gut axis is composed of the gut and the brain through the neurological, immunological, and endocrine systems, and the gut flora can use the “gut–brain axis” as a bridge to establish a connection with sleep, which mainly includes the immunological pathway, the neurological pathway and the endocrine pathway.

A study found that increasing DF intake in daily meal structure benefitted sleep disorders. This is because DF is metabolized in the colon, increasing the variety and number of beneficial bacteria and their SCFAs in the gut, which leads to improvement of the damaged intestinal barrier, stimulating the secretion of sleep cytokines, inhibiting inflammatory pathways, and increasing serotonin secretion, which further leads to the regulation of sleep disorders. Butyric acid is one of the SCFAs that are intestinal metabolites, a natural product of the bacterial fermentation of DF in the colon. Studies have found that butyric acid has great potential value in the neuroprotection of the brain [73]. DF has also been found to be potentially relevant to the effective prevention of dementia and Alzheimer’s disease in older people, preventing the development of disordered microbiomes and associated cognitive dysfunction in people at risk of developing Alzheimer’s disease [74].

### 6.6. Other Health Effects of DF

In addition to the above-mentioned health effects, DF also has some other beneficial health effects on a wide range. It is thought to have some potential benefits in preventing and alleviating depression in adults [75]. The supplementation of hypertensive patients with a certain amount of DF supplements may improve depression and anxiety through the gut–brain axis by increasing the beneficial components of the intestinal flora, such as SCFA, bifidobacteria, and spirochetes [76]. Increased intake of fiber-rich foods has also been predicted to relieve severe headaches or migraines [77].

DF may be effective in improving bruxism in young people [78] and reducing inflammatory conditions such as periodontitis [79]. DF improves cutaneous wound healing and scar formation via the metabolite-sensing receptor GPR43 [80]. A comprehensive assessment of the association between DF intake and gallstone disease (GSD) found that higher DF intake was significantly associated with a reduced risk of GSD [81]. Since different fiber types from different sources have distinct properties, the data on their efficacies and functionalities are in a very narrow range, which need to be explored further. In summary, DF has a variety of physiological effects and plays a probiotic role in human health, so the intake of foods high in DF may prevent or reduce the risk of developing many diseases.

## 7. DF Applications and Future Perspectives in the Food and Other Industries

**Figure 5:**
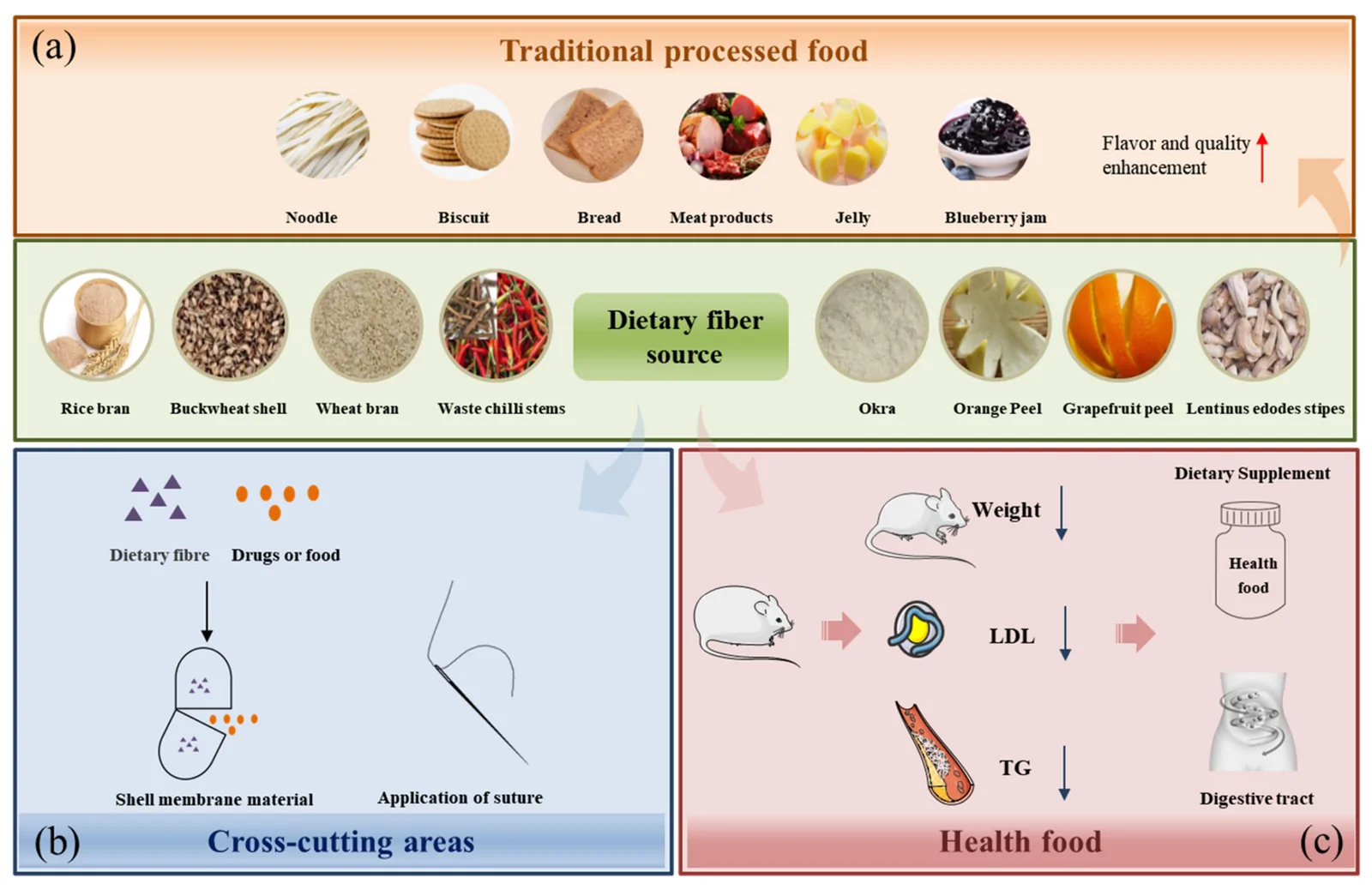
Overview of dietary fiber extracted from wastes in several food-related applications. (**a**) Application of waste dietary fibers in different traditional processing systems. (**b**) Application of waste dietary fibers in cross-cutting fields. (**c**) Development of related dietary fiber health products based on the nutritional functions of dietary fibers. Red upward arrows represent promotion or enhancement effects, while blue downward arrows represent inhibition or reduction effects.

By-products of food processing, such as seeds, skins, pomace, and shells, are often a good source of DF, and some skins, in particular, are rich in IDF. The addition of DF to foods also optimizes a wide range of physicochemical properties and enhances the quality of the food, a practice that has gained popularity in recent years. Based on the excellent quality of dietary fibers and their nutritional and functional properties, more and more dietary fibers from wastes have been gradually developed and utilized and have achieved better application prospects in many fields, such as in traditional foods, health foods, and their applications in cross-cutting areas. Figure 5 summarizes the application profile of dietary fibers from waste in several food-related fields.

### 7.1. Traditional Processed Foods

Currently, in the processing of traditional foods, based on the physical properties of their DF (e.g., water-holding, oil-holding, swelling, viscosity, etc.), the addition of high DF can enhance a variety of physicochemical properties of the food, and comprehensively achieve product quality enhancement, nutritional and functional strengthening. Table 2 summarizes the applications of dietary fibers from waste in traditional food processing and their gained quality effects.

#### 7.1.1. Flour-Based Products

DF can interact with starch, protein, moisture, and other components in food to further enhance the water retention capacity and oil-holding capacity of the product, improve the hardness and elasticity of the pasta product, and make it have better texture characteristics, thus improving the quality of the pasta product. Additional DF additions can make pasta products exhibit more complex and looser microstructures. These additions of DF can increase the complexity of the starch–protein network, forming an interwoven mesh in the dough and enhancing dough stability [90]. At the same time, DF promotes the binding of starch to water in pasta products, thus, improving the food’s ability to bind with water during processing and improving the quality of the product. Most SDFs can interact non-covalently with gluten protein through hydrogen bonding and hydrophobic interactions [90]. Adding DF to processed foods can enhance their product quality and nutritional value. For example, a study found that adding IDF to Mantou decreased the Zeta potential of gluten in the proofing and decreased cooking stages by 46.64% and 68.10%, respectively, indicating that IDF could promote gluten aggregation by reducing electrostatic repulsion [91]. The addition of bran IDF to pasta can improve some of the nutritional value of the pasta and effectively extend the shelf life of the pasta. In particular, the addition of low concentrations of IDF can improve the chewiness and disulfide bond content of noodles, which is conducive to improving the quality of noodles. IDF extracted from buckwheat hulls is added to noodles or dough after ultra-fine grinding to simultaneously increase the rigidity, viscoelasticity, and resistance to deformation of proteins, improving the texture of noodles [82]. Pumpkin seeds and soya dregs (from soya milk and tofu processing) are by-products of food processing and are rarely utilized. Both pumpkin seeds and soya dregs contain high levels of DF and can be added to food products as cheap potential DF resources. The application of pumpkin seeds and soya dregs in the production of gluten-free proteins and fiber-rich pasta can increase the added value of the waste [83]. Yao et al. proposed a method to modify the structural properties of soya bean dregs DF using a combination of ultrasound and high-speed shear treatments. Adding modified soya dregs DF to biscuits not only preserved the flavor but also increased the DF content of the food. This method also led to the utilization of soybean processing waste by-products and contributed to global green environmental benefits [84]. The addition of IDF from feijoa (Acca sellowiana) to wheat bread significantly increased the total phenolic and flavonoid content and antioxidant activity of the product and improved the flavor of the bread samples, but when added at levels greater than 2%, it led to undesirable flavor and texture in the pieces of bread [92]. Baking barley and oatmeal as natural sources of β-glucan improves the organoleptic qualities of foods [93]. However, excessive addition of dietary fiber can lead to dilution of the gluten network structure and may also harm the food quality; therefore, the addition of different dietary fiber classes or varieties requires further experiments to confirm the appropriate amount of dietary fiber to be added.

#### 7.1.2. Dairy and Meat Products

A study has found that a combination of binary probiotics and goji dietary fiber (WDF) in yogurt significantly improves yogurt quality and product value [86]. The addition of IDF from shiitake mushroom stalks to meat products promotes the formation of a compact gel network structure and improves the quality of meat products [33]. The addition of apple peel DF to lean turkey meat improves meat firmness and reduces cooking losses [94]. Potato DF has a significant ameliorating effect on the myofibrillar protein gel properties of chicken patties, resulting in the improvement of processed meat products [87]. Inulin SDF from blue agave was used for the enhancement of balsamic sausages [95]. The application of DF extracted from waste in meat and dairy products is still in the developmental stage. Not all DF are expected to optimize the variety of dairy and meat products due to the large functional differences, like different DF.

Therefore, more experimental studies are needed to determine which class of DF positively contributes to certain properties. As the current data only examines some of the characteristic parameters of the products, more in-depth studies are needed to determine the positive effects of DF in dairy and meat products.

#### 7.1.3. Other Foodstuffs

Steam blasting and enzymatic hydrolysis were used to treat the DF in soya bean dregs, leading to an increase in its SDF content, which, when applied to tofu production, was found to improve the firmness and viscoelasticity of the bean curd [37]. Dietary fiber from date fruit pomace (DFP) can be used as a dietary supplement and produced as a high-value-added product through fermentation [24]. SDF obtained from navel orange peel by mixed solid state fermentation (M-SDF) is added to the jelly to help maintain the texture of the jelly with better firmness, viscosity, and chewiness [88]. High internal phase emulsion gels (HIPE-Gels) and oleo gels were successfully prepared by a synergistic combination of natural triterpenoid soap tree saponins (QSs) and citrus dietary fiber (CDF), which can transform the structure of edible liquid oils into healthy gels for substituting saturated and trans fats in foods [96]. A combination of microwave and enzymatic methods was used to modify grapefruit peel insoluble dietary fiber (GP-IDF) to obtain SDF, and its (GP-IDF-SDF) addition to jam products improved their color, texture, spreadability, and shelf life [89].

Currently, research and development on DF from waste by-products focuses more on pasta, dairy, and meat products. There are fewer cases of research on the application of DF from waste by-products in other food products. However, DF from waste by-products has many excellent characteristics and beneficial properties. Looking ahead, the research and development and application of dietary fiber from waste should be increased to enrich the types of high DF foods and increase their application in various types of foods, so as to meet the growing demand for various special foods and to make greater resource reuse of food waste. At the same time, it can also alleviate, to a greater extent, the troubles brought about by unhealthy diets, such as obesity, and enhance people’s physical fitness.

### 7.2. Application of DF in Functional (Health) Foods

Based on the nutritional and functional properties of dietary fiber, many researchers and scholars have considered the application of waste dietary fiber in the development of functional health food. Its application also covers many fields such as functional ingredient enhancement, product structure improvement, and targeted delivery, etc. Currently, the wider application is to focus on its high-fiber nutritional value, and add DF to food as a nutrient-enhancing substance and dietary supplement []. In particular, the development of dietary fiber lipid-lowering health products has been confirmed by many studies to have excellent efficacy in weight management and metabolic regulation. It can produce SCFAs in the human body, enhance the sense of satiety, improve blood lipid parameters, regulate the expression level of genes related to lipid synthesis, and thus inhibit cellular and fat synthesis, to achieve the efficacy of lipid-lowering and weight loss [97]. For example, low-sugar noodles rich in high DF significantly reduced body weight, percentage of fat, total cholesterol, LDL, cholesterol, etc., in the animal model [98]. DF is also highly beneficial for diabetic patients, as it attenuates postprandial glucose release and alleviates metabolic stress [99,100]. Whole wheat cookies enriched with capsicum stem nanocellulose significantly reduced body weight gain in mice in animal experiments with waste capsicum stem DF (nanofibrillar cellulose extracted from capsicum stems) [85]. In addition, the intake of foods with high DF content can prevent or reduce the risk of many diseases. Therefore, dietary fiber can be added as a nutritional alternative to healthy foods for people with chronic diseases, such as diabetics and hyperlipidemic patients, who need to strictly prevent and control the intake of sugar. Bran IDF is effective in promoting intestinal motility, increasing satiety, and lowering postprandial blood sugar [101]. Wheat bread, which is rich in insoluble dietary fiber from ginseng pomace, has also been shown to lower the glycemic index [102]. Although dietary fiber exhibits vast potential for functional product development owing to its diverse physiological activities, current research and development efforts remain restricted, resulting in a narrow product spectrum dominated by lipid-lowering formulations. For example, dietary fiber also has good potential for development in the management of intestinal health, intestinal barrier repair, and immunomodulation. For example, dietary fiber extracted from mango peel has been shown to have therapeutic potential for improving intestinal health [103]. Furthermore, the research and development of foods for special medical purposes (FSMPs), targeting the adsorption of harmful substances, remain relatively scarce. However, the market development for this and other health products is scarce, and most basic research has not yet been translated. Therefore, investment should be further increased to develop functional products in more directions, as well as composite products with diversified efficacy.

### 7.3. Cross-Cutting or Other

Due to its natural structural properties, such as high adsorption, biocompatibility, and degradability, dietary fiber also shows diversified application potentials in cross-cutting fields such as medicine, agriculture, environmental protection, materials, and daily chemicals. In particular, the use of dietary fibers as carriers for nutrient or drug delivery in the material, food, or pharmaceutical industries makes them potential materials for food and biomedical applications. For example, in the food industry, a composite shell material developed using peppercorn seed soluble dietary fibers and soybean isolate proteins can be used to deliver peppercorn essential oils, which are encapsulated to not only enhance the antimicrobial activity but also retain antioxidant activity [38]. Because of the fiber-stabilized oil-in-water emulsion properties, nano-primary fibrillated cellulose oil-in-water Pickering emulsions can be used for the encapsulation and delivery of vitamin D3 [104]. The use of dietary fiber in microencapsulated material applications not only serves as a wall material but also has the advantage of increasing the dietary fiber content of the human diet [105]. In the field of pharmaceutical engineering, there are also many applications of dietary fiber as a drug delivery wall material in research and development, such as the use of synthetic materials containing dietary fiber as a sustained drug delivery carrier for the relief of colonic inflammation [106]. Singh et al. designed pH-responsive dietary fiber hydrogels based on Azadirachta indica gum as sustained drug delivery carriers for methylprednisolone to treat inflammatory colon diseases [107].

In addition to being used as embedding and delivery encapsulation materials, dietary fibers can be used as absorbable biomaterials in a variety of applications. Wei, C et al. found that Ramie fibers had good hygroscopicity and blood compatibility and thus had potential applications in medical dressings [108]. The significant antibacterial activity of Ramie sutures made from Ramie plant fibers against Escherichia coli, Bacillus subtilis, and Staphylococcus aureus can be attributed to the inherent bacteriostatic ability of Ramie plant fibers, and the wound healing ability of Ramie sutures was also demonstrated to be comparable to that of commercially available BMSF sutures in terms of biocompatibility and wound closure efficacy [109]. A fiber–biopolymer composite patch prepared from discarded plant fiber banana pseudostem using chitosan and guar gum was found to have good antimicrobial properties and could be developed as an alternative wound healing material [110]. Simple recycling of waste cellulose and alginate fibers was found to be used as fireproofing and EMI shielding composites [111].This study also confirms the possibility of dietary fiber as a material in areas other than medicine. In addition to the above application directions, many functional properties of dietary fibers are still to be further developed, such as the development of feed additives and supplements based on the nutritional value of dietary fibers; the development of adsorbents for wastewater treatment based on the strong adsorption capacity of dietary fibers for heavy metals (e.g., Cu^2+^, Pb^2+^); and the development of cosmetic additives based on the antioxidant effects of dietary fibers.

### 7.4. DF Future Perspectives

With the continuous increase in people’s emphasis on sustainable development, converting dietary fiber by-products into high-value-added products through biological and chemical means has become a key way to achieve resource recycling and the upgrading of the health industry. In the future, the exploration and research of dietary fiber by-products should mainly focus on the innovation of extraction technology and the progress of application.

In extraction technology innovation, the future will be more inclined to the green, efficient new technology of extracting dietary fiber. Physical techniques such as high-voltage pulsed electric fields and microwave-assisted extraction, as well as enzymatic hydrolysis and microbial fermentation, can achieve efficient extraction of dietary fiber while retaining its functional characteristics to the greatest extent. In addition, the introduction of nanotechnology and supramolecular assembly technology further enhances and improves the biological activity and physicochemical properties of dietary fiber, opening up new paths for its application in high-end fields such as pharmaceutical carriers and smart foods.

For the development of dietary fiber applications in food, beyond conventional functional foods, future work shall focus on personalized nutrition for specific populations and the creation of beneficial functional products tailored to different groups. In the field of medicine, modified dietary fiber can be used as a sustained-release carrier for drugs to enhance their efficacy and safety. In the field of environmental protection, dietary fiber by-products can be used to prepare degradable packaging materials, heavy metal adsorbents, etc., which helps to solve environmental pollution problems.

However, the current dietary fiber waste still faces a shortage of technology and high costs, and the industrialization scale, such as basic theory research, is a major bottleneck. Overall, in the future, it is necessary to enhance interdisciplinary research, conduct in-depth studies and analyses of the structure–activity relationship of dietary fiber, reduce production costs, and accelerate the transformation of technological achievements. The transformation of dietary fiber from waste to treasure will become an important engine for achieving efficient resource utilization and promoting the sustainable development of the health industry.

## 8. Conclusions

DF, as the “seventh-largest nutrient”, has a wide range of health benefits in improving gut microbiota, reducing the risk of obesity and cardiovascular disease, and regulating gut health. Although most agricultural by-products contain high levels of DF, DF resources in current agricultural processing by-products are being inefficiently utilized. Waste dietary fiber has shown great potential in the fields of traditional food improvement, functional health food development, and the cross-border integration of food, medicine, and materials. Dietary fiber can achieve the synergistic improvement of traditional food texture and flavor through molecular modification technology; construct probiotic functional systems with polyphenols, probiotics, etc.; and demonstrate unique advantages and potential in multiple fields such as edible packaging, delivery systems, and application materials. However, the current exploration of the potential application of dietary fiber is not deep enough, and many research results still face severe challenges in terms of transformation, especially regarding the structural differences, physicochemical properties, and complex mechanisms of action of dietary fiber from different sources in human metabolism, which are not yet fully understood. In the future, we should increase the deep exploration of the potential application of waste dietary fiber, and at the same time, deeply analyze the structure–activity relationship and metabolic mechanism of dietary fiber, truly realizing the value leap from food processing waste to health functional factors, laying the foundation for the development of the food industry and sustainable utilization of resources.

## Figures and Tables

**Table 1:** Positive effects of different sources of dietary fiber on human health or disease.

Physiological Functions	Sources	DF	Research Outcomes	References
Colitis	Okara	IDF	Improved colonic environmental disorders caused by acute ulcerative colitis and affected the proliferation of key bacteria involved in the SCFA synthesis pathway.	[26]
	Barley leaf	IDF	Modulated gut microbiota composition in DSS-induced mice, with reduced decreases in SCFAs and secondary bile acids.	[47]
	Rice Hull	IDF	Suppressed the colonic TLR4/MyD88/NF-κB signaling pathway and inhibited the transcription of inflammatory cytokines.	[48]
	Psyllium	DF	Induced the expression of genes involved in bile acid (BA) secretion, increased fecal BA levels, and activated farnesoid X receptor (FXR) signaling. FXR knockout experiments further suggested that FXR activation played a central role in alleviating colitis.	[49]
	Bamboo shoot	DF	Alleviated colonic inflammation mainly by increasing the relative abundance of beneficial bacteria (Alistipes and Lactobacillus) while decreasing the abundance of pathogenic bacteria (Escherichia-Shigella, Enterococcus, and Proteus).	[50]
	Lentinula edodes byproducts	SDF	Clinical symptoms were alleviated by maintaining the Th17/Treg balance, increasing immunoglobulin A levels, reducing tumor necrosis factor-α production, and promoting Foxp3 and vitamin D receptor mRNA expression.	[51]
Diabetes	Bergamot	SDF/IDF	Regulated glucose tolerance and blood glucose levels, and reduced serum total cholesterol, triglycerides, and low-density lipoprotein levels.	[52]
	Konjac	SDF	Significantly reduced lipid profiles, including TC, TG, VLDL, and LDL levels, improved related metabolic parameters, and regulated key metabolic proteins, including PPAR-γ and p-SREBP-1C.	[53]
Obesity	Okara	IDF	Significantly ameliorated body weight gain, fat accumulation, dyslipidemia, and steatosis, and improved the levels of acyl-coenzyme A oxidase 1 (ACOX1), malonyl-CoA, acetyl-CoA synthase (ACS), acetyl-CoA carboxylase (ACC), and carnitine palmitoyltransferase-1 (CPT-1).	[54]
	Pomelo peel	SDF/IDF	Significantly reduced body weight gain and fat accumulation in liver and epididymal tissues, alleviated HFD-induced dyslipidemia, and restored triglyceride, low-density lipoprotein cholesterol, and high-density lipoprotein cholesterol levels to normal levels.	[55]
	Bamboo Shoot	SDF	Effectively reduced lipid accumulation in adipose tissue, alleviated dyslipidemia and insulin resistance, improved metabolic disorders by modulating PPAR-related fatty acid pathways, and significantly enriched beneficial bacteria such as Bifidobacterium, Akkermansia, Dubosiella, and Alloprevotella.	[56]
Hyperlipidemia	Tremella fuciformis residues	SDF/IDF	Reduced total cholesterol (TC), triglycerides (TGs), and low-density lipoprotein cholesterol (LDL-C) levels and increased high-density lipoprotein cholesterol (HDL-C) levels.	[57]
	Shatianyu	DF	Regulation of lipid metabolism by modulating microbiota-derived butyric acid production to synergistically alleviate hyperlipidemia.	[29]
	Bergamot-derived	SDF/IDF	Inhibited increases in TG, TC, and LDL-C levels induced by a high-fat diet, improved liver pathological abnormalities, upregulated LXRα and CYP7A1 expression, and downregulated SREBP-1c, FAS, ACC, SREBP-2, PGC-1α, PRDM16, UCP-1, and PPARγ expression in brown adipose tissue.	[58]
Cholesterol	Tea	DF	Reduced hepatic index, serum and hepatic total cholesterol (TC), triglycerides and LDL cholesterol, increased serum and hepatic HDL cholesterol to total cholesterol ratios, facilitated fecal lipid excretion, significantly increased serum and hepatic superoxide dismutase and glutathione peroxidase activities, and reduced lipid peroxidation.	[59]
	Black Rice	DF	Hypocholesterolemic effect through cholesterol adsorption, improved lipid distribution, cholesterol metabolism regulation and intestinal flora regulation.	[60]

**Table 2:** Positive effects of dietary fiber from different waste sources in food processing.

Product Category	Source (Waste or By-Products)	Food Applications	Optimization Results	References
Pasta products	Buckwheat Hull	noodle	Increased viscoelasticity and resistance to deformation of the dough, increased starch pasting rate and reduced starch thermogel stability, as well as increased rigidity of the protein structure (β-folding), resulting in improved noodle texture.	[82]
	Okara and Pumpkin Seeds	noodle	Enhanced the nutritional value of noodle products and improved the utilization of raw material waste.	[83]
	Okara	cookies	Biscuits made with 6% of modified soya bean dregs fiber when added to the dough showed the best printing properties; it also improved the utilization of the raw material waste soya bean residue and increased the dietary fiber content of the biscuits.	[84]
	Waste Chili Stems	biscuits	The amount of nanocellulose (7%) allowed the preparation of biscuits of acceptable overall quality, with the corresponding biscuits having a regular appearance and a relatively smooth surface. It was found that consumption of biscuits containing nanocellulose significantly reduced food intake and inhibited weight gain in mice through experimental animal studies in mice.	[85]
Dairy meat products	Wolfberry	Yogurt	Could significantly improve yogurt quality and product value.	[86]
	Lentinus Edodes Stipes	meat product	Promoted the formation of ionic and hydrogen bonds, leading to the formation of a compact gel network structure that trapped more water molecules in the gel, thereby improving the quality of the meat product.	[33]
	Potato	chicken patty	Reduced α-helix content and increased β-fold content in samples. Significantly improved myofibrillar protein gel characteristics and enhances the quality of processed meat products.	[87]
Other food stuffs	Navel Orange Peel	Jelly	Jellies made by blending solid fermented SDF exhibited a homogeneous porous mesh structure, which helped to maintain the texture of the jelly.	[88]
	Grapefruit Peel	jam	Improved color, texture and spreadability of jams with pectin; improved stability of jams.	[89]

## Data Availability

The original contributions presented in this study are included in the article. Further inquiries can be directed to the corresponding authors.
